# Phase I study of NK105, a nanomicellar paclitaxel formulation, administered on a weekly schedule in patients with solid tumors

**DOI:** 10.1007/s10637-016-0381-4

**Published:** 2016-09-05

**Authors:** Hirofumi Mukai, Ken Kato, Taito Esaki, Shouzou Ohsumi, Yasuo Hozomi, Nobuaki Matsubara, Tetsuya Hamaguchi, Yasuhiro Matsumura, Rika Goda, Takayuki Hirai, Yoshihiro Nambu

**Affiliations:** 1 Department of Breast and Medical Oncology, National Cancer Center Hospital East, 6-5-1, Kashiwanoha, Kashiwa, Chiba, 277-8577 Japan; 2 Gastrointestinal Medical Oncology Division, National Cancer Center Hospital, National Cancer Center, Tokyo, Japan; 3 Department of Gastrointestinal and Medical Oncology, National Hospital Organization Kyushu Cancer Center, Fukuoka, Japan; 4 Department of Breast Surgery, National Hospital Organization Shikoku Cancer Center, Ehime, Japan; 5 Department of Breast and Endocrine Surgery, University of Tsukuba Hospital, Ibaragi, Japan; 6 Division of Developmental Therapeutics, National Cancer Center Hospital East, Chiba, Japan; 7 Nippon Kayaku Co., Ltd., Tokyo, Japan

**Keywords:** NK105, Paclitaxel, Polymeric micelles, DDS, Breast cancer

## Abstract

Previous studies have established the rationale for NK105, a nanomicellar formulation of paclitaxel, administered every 3 weeks. The aim of this phase I study was to determine the recommended dose and pharmacokinetics of weekly administered NK105. NK105 was administered by a 30-min infusion once weekly for three consecutive weeks in each 4-week cycle. In the dose-escalation phase, three to seven patients with solid tumors were enrolled to each of the four dose levels (50–100 mg/m^2^; *n* = 16). At a dose level of 100 mg/m^2^, predefined dose-limiting toxicity (DLT) manifested in only one out of six evaluable patients, whereas a dose delay due to neutropenia during the first course occurred two patients. None of the three patients given 80 mg/m^2^ had a dose reduction, while a dose delay occurred in two. NK105 exhibited linear pharmacokinetics at doses of 50–100 mg/m^2^, and approximately 5 % of total paclitaxel was released from micelles. Thus, the recommended dose was set at 80 mg/m^2^, and an additional 10 advanced breast cancer (ABC) patients were given this dose in the dose-expansion phase. DLT manifested in two patients, and grade ≥ 3 neutropenia was found in eight patients. Among the nine patients who completed the first cycle, four had a dose reduction, mostly because of neutropenia. Of the 10 patients, six achieved partial response (PR), and four achieved stable disease (SD) status. Overall, weekly NK105 was well tolerated and had a desirable antitumor activity profile. Further investigations of NK105 in ABC patients are currently underway.

**Electronic supplementary material:**

The online version of this article (doi:10.1007/s10637-016-0381-4) contains supplementary material, which is available to authorized users.

## Introduction

The antimicrotubule agent paclitaxel (PTX) has a broad spectrum of antitumor activity against different types of solid tumors, including ovarian, breast, stomach, lung, and head and neck cancers [1–3]. NK105 is a PTX-incorporating “core-shell-type” polymeric micellar nanoparticle formulation that can be administered intravenously without the use of polyoxyethylene hydrogenated castor oil (Cremophor EL) or ethanol as a vehicle [4]. Solid tumors have unique characteristics, such as hypervascularization, presence of vascular permeability factors stimulating extravasation within cancer, and reduced lymphatic clearance of macromolecules that collectively underlie the enhanced permeability and retention (EPR) effect [5]. Macromolecular micellar formulations such as NK105, developed on the basis of the EPR effect, aim to support the tumoritropic delivery of a drug as well as its sustained retention and direct antitumor effect in the cancer tissue [6, 7].

In vivo, NK105 exerted a significantly more potent antitumor effect than free PTX, probably because of the enhanced tumor exposure due to the EPR effect [4]. In a previous phase I study, NK105 was administered intravenously, without antiallergic premedication on a tri-weekly schedule to patients with solid malignancies. Its dose-limiting toxicity (DLT) was reflected in neutropenia, therefore 180 mg/m^2^ every 3 weeks was designated as the maximum tolerated dose (MTD). Its recommended dose was determined to be 150 mg/m^2^ every 3 weeks, at which its area under the plasma concentration versus time curve (AUC) was more than 15 times greater than that of the conventional PTX formulation (210 mg/m^2^) [8]. In our subsequent phase II study, we found that NK105 administered at 150 mg/m^2^ every 3 weeks had a favorable safety profile and activity, comparable to those of the conventional PTX formulation in previously treated patients with advanced or recurrent gastric cancer [9]. Thus, previous clinical studies of NK105 administered on a tri-weekly schedule have provided a proof of concept for this nanoparticle micellar formulation of PTX as a better alternative to the conventional formulation with regard to the efficacy, safety, and convenience in dosing.

Main recommendations concerning chemotherapy for advanced or metastatic breast cancer include the sequential use of single chemotherapeutic agents. Although taxanes can be used as the first-line therapy, they have not shown superior benefits to anthracyclines [10]. PTX has been approved for breast cancer in various settings with both tri-weekly and weekly regimens in Japan. In a phase III randomized controlled study, weekly PTX (80 mg/m^2^) treatment was shown to be more effective than tri-weekly administration of 175 mg/m^2^ for metastatic breast cancer in terms of the tumor response and patient survival [11]. Based on this finding, it was presumed that NK105 could be administered on the weekly dosing schedule, and this might lead to a better tumor response than the tri-weekly administration. In the present phase I study, we examined the safety, pharmacokinetics (PK), and efficacy of NK105 administered on a weekly schedule in patients with solid tumors to determine its recommended weekly dose. We also evaluated the safety and preliminary efficacy of NK105 administered at its recommended weekly dose in patients with advanced breast cancer.

## Methods

This study consisted of two parts. Part I was the dose-escalation phase designed to determine the recommended dose of NK105 by evaluating its safety and tolerability at each dose level. Part II was the exploratory dose-expansion phase designed to evaluate the safety and preliminary efficacy of NK105 at its recommended weekly dose determined in Part I in an additional cohort of breast cancer patients. Part I was carried out at the National Cancer Center Hospital and National Cancer Center Hospital East. In addition to these two institutions, three additional sites (National Hospital Organization Kyushu Cancer Center, National Hospital Organization Shikoku Cancer Center, and Jichi Medical University Hospital) were included in Part II. This study was registered with the Japan Pharmaceutical Information Center (JAPIC) Clinical Trials Information (www.clinicaltrials.jp; study identifier: JapicCTI-101,233).

### Patients

Patients with histologically or cytologically diagnosed solid tumors refractory to standard treatment or for whom no effective treatment was available were eligible for Part I of this study, provided that they met the following criteria: age 20–75 years; ECOG performance status of 0 to 2; maintenance of adequate organ function; normal hematopoietic (WBC 4000–10,000/mm^3^, ANC ≥2000/mm^3^, platelet count ≥100,000/mm^3^, hemoglobin ≥9.0 g/dL), hepatic (AST and ALT ≤2.5 times the upper limit of normal, or ≤5 times the upper limit of normal in the presence of hepatic involvement, total bilirubin ≤1.5 mg/dL) and renal functions (serum creatinine ≤1.5 mg/dL). Key exclusion criteria included grade ≥ 2 peripheral sensory neuropathy. To enter Part II of this study, patients had to meet these criteria as well as to have advanced or recurrent breast cancer diagnosed histologically or cytologically and at least one measurable tumor lesion as outlined by the New Response Evaluation Criteria in Solid Tumors (RECIST) guideline version 1.1. Patients were not considered for enrollment in Part II if they had received any taxane against advanced breast cancer or postoperative adjuvant therapy during the previous 6 months.

### Treatment

#### Study drug and its administration

NK105 was supplied by Nippon Kayaku Co., Ltd. (Tokyo, Japan) in glass vials containing a dose equivalent to 100 mg of PTX per vial. Each dose solution of NK105 was prepared in 100 mL of a 5 % glucose solution for injection and administered intravenously over about 30 min at a speed of about 200 mL/h. Any premedication was not required before the study drug administration.

#### Dosage and dose escalation schedule

NK105 was administered once-weekly (at intervals of ≥7 days) for three consecutive weeks (days 1, 8, and 15), followed by a one-week (day 22) rest. This four-week cycle was repeated until disease progression or unacceptable toxicity. In Part I, the starting dose of NK105 was 50 mg/m^2^ (level 1), which was then escalated stepwise to 65 (level 2), 80 (level 3), 100 (level 4), and 120 mg/m^2^ (level 5) until its recommended dose was determined. Dose escalation to the next higher level was allowed if none of the first three patients treated at a current level experienced any DLT (defined below) during the first cycle. If one of the first three patients experienced any DLT during the first cycle, three more patients were enrolled at the level. Dose escalation was also allowed if only one of the six patients treated at the level experienced any DLT during the first cycle.

#### Dosage modifications

In both Parts I and II, patients were withheld from starting a new cycle until recovery to grade 1 neutropenia, thrombocytopenia, and non-hematologic toxicities (or non-hematologic toxicities of an equal or lower grade than baseline). During a cycle, patients were to suspend (delay) each dose scheduled until recovery from grade 2 neutropenia, thrombocytopenia, and non-hematologic toxicities. If patients experienced any DLT or had to delay their dose(s) by ≥8 cumulative days during a cycle or were unable to start a new cycle even at 35 days after starting the current cycle, they had to reduce their doses for the next cycle by one level.

#### Concomitant treatments

During the study, patients were not permitted to concomitantly receive any therapy for the malignancy, or any other investigational drug. Granulocyte-colony stimulating factor (G-CSF) support was allowed in the second and subsequent cycles or after grade 4 neutropenia lasting for 5 days had been identified.

### DLT and recommended dose determination

A DLT was defined as any of the following toxicities occurring during the first cycle and assessed as definitely or probably related to NK105: 1) grade 4 neutropenia lasting for ≥5 days; 2) grade 4 thrombocytopenia; and 3) grade ≥ 3 non-hematologic toxicities. If two or more of the first three patients or two or more of the total six patients treated at a dose level experienced any DLT, the dose was to be designated as the MTD for the weekly administration of NK105. To determine the recommended dose for Part II, six patients in total were treated at one level lower than the MTD and subsequently evaluated. The final decision on the recommended dose was made by consulting with an independent data monitoring committee.

### Follow-up and evaluation

#### Safety

Patients underwent a physical examination and routine laboratory tests once a week and electrocardiography (ECG) once per cycle to confirm their safety and to detect adverse events (AEs). AEs were graded according to the Common Terminology Criteria for Adverse Events (CTCAE) version 4.0, and their causal relationships with the study drug were evaluated by the investigators.

#### Pharmacokinetics

Plasma concentrations of released PTX and total PTX (both micelle-incorporated and released) were measured in all patients enrolled in Part I. For the measurement, blood was collected before as well as at 15 min, 30 min (at the end of infusion), 1 h, 3 h, 6 h, 24 h, 72 h, and 168 h after the start of the first dose of the first cycle. Blood collection was also done pre-dose, at the end of the infusion and at 24 h after the start of the third dose of the first cycle, and the first dose of the second cycle. The plasma concentrations of total PTX were determined by liquid chromatography/tandem mass spectrometry (LC-MS/MS) as described previously [8]. The plasma concentrations of released PTX were estimated by the equilibrium dialysis. Briefly, the PTX levels in the dialysis outer and inner liquids were measured by the above-mentioned LC-MS/MS assay and used to estimate the proportion of the protein-unbound PTX relative to total PTX in the plasma. This proportion and the plasma total PTX concentration measured without equilibrium dialysis were used to calculate the plasma concentration of the protein-unbound PTX. This value was substituted for the “plasma concentration of the protein-unbound PTX” in the previously obtained correlation equation for predicting the plasma concentration of the released PTX. Plasma PTX concentrations were summarized for each dose level. The following PK parameters of total PTX were calculated for each patient using a non-compartmental model by using the WinNonlin software (Professional Edition version 5.2.1 or 6.1, Pharsight Corporation, Mountain View, CA, USA): the maximum observed plasma concentration (C_max_); time to C_max_ (T_max_); AUC from time zero to infinity (AUC_0-inf_); total clearance (CL_tot_); volume of distribution in the steady state (V_ss_); mean residence time from time zero to infinity (MRT_0-inf_); and half-life of the terminal elimination phase (t_1/2_). For the released PTX, the same PK parameters except for CL_tot_ and V_ss_ were calculated. From the AUC_0-inf_ values of total and released PTX, the proportion of released relative to total PTX in the plasma was calculated.

#### Tumor response

A computed tomography (CT) examination was performed once per cycle. Tumor response was evaluated according to the RECIST guideline version 1.1. The objective response rate (ORR) was calculated as the percentage of patients who achieved the best overall complete (CR) or partial (PR) response.

### Statistical analyses

The Safety Analysis Set was defined as patients who received the study drug administration. The PK Analysis Set was defined as a subset of the Safety Analysis Set that excluded patients without adequate data for PK analysis. The efficacy analysis set was defined as patients who underwent the efficacy assessment. The first safety and efficacy analyses were performed at three months after initiating the study drug administration to the last patient. Then, the data were finally updated when all subjects completed the study.

AEs reported from all patients treated with NK105 were coded using the Medical Dictionary for Regulatory Activities (MedDRA) version 16.1 and summarized by the preferred term and system organ class. Confidence intervals (CIs) for binary endpoints were calculated using the method of Clopper and Pearson [12, 13].

## Results

### Disposition of subjects

During the period from August 27, 2010, to November 4, 2011, 26 eligible patients were enrolled, including 16 in Part I and 10 in Part II (Table 1). All enrolled patients received NK105 at least once. In Part I, one patient allocated to level 4 (100 mg/m^2^) developed a grade 2 infusion reaction and was excluded from the study immediately after the start of the first infusion. Therefore, the patient could not undergo the efficacy assessment and blood collection for the PK study. Thus, the patient was excluded from the PK and efficacy analysis sets.

**Table 1:** Patient characteristics

	Dose-escalation phase	Dose-expansion phase
No. of patients	16	10
Gender		
Male	10	-
Female	6	10
Age (years)		
Median	66.0	61.0
Range	46–74	41–68
ECOG PS		
0	5	7
1	11	3
Primary tumor		
Gastric	2	-
Esophageal	4	-
Esophageal, oral floor	1	-
Renal pelvis	1	-
Prostate	1	-
Bladder	1	-
Breast	4	10
Occult primary	2	-
Primary or recurrent		
Primary	6	2
Recurrent	10	8
No. of prior chemotherapy regimens		
0	0	2
1	5	3
2	5	5
≥ 3	6	0

### Recommended dose determination

In Part I, the dose of NK105 was escalated from level 1 (50 mg/m^2^) up to level 4 (100 mg/m^2^). At level 4, one of the first three patients had an unacceptably long delay of the study drug treatment for 11 days in total due to persistent grade 3 neutropenia and later terminated the treatment. Of the four other patients enrolled at this level, one developed an infusion reaction (flushed face and chest, dyspnea) in two minutes and discontinued the treatment immediately after the start of the first infusion. This patient was excluded from the recommended dose determination because the event was thought to be a dose-independent reaction. Of the remaining six patients, one experienced grade 4 neutropenia lasting for ≥5 days. Four patients had to delay their dose(s) and two patients had to reduce their dose(s) due to treatment-related AEs during the first cycle.

The protocol permitted a further dose escalation to level 5 (120 mg/m^2^) because only one of the six patients treated at level 4 experienced a DLT. On one hand, treatment-related AEs during the first cycle led to at least one dose delay in four patients and to a dose reduction in two patients at level 4. On the other hand, none of the three patients at level 3 (80 mg/m^2^) experienced any treatment-related AE that led to a dose reduction during the first cycle. Although the AEs leading to dose reductions did not meet the criteria for DLTs, it was concluded that for assessing the tolerability of weekly administration of NK105 and determining its recommended dose, the frequent need of dose reductions or dose delays should be taken into as much consideration as DLTs. Through the consultation with an independent data monitoring committee, the weekly dose of NK105 that could be administered safely for more than one cycle was determined to be 80 mg/m^2^.

In Part II, all 10 patients enrolled were treated with NK105 at a dose of 80 mg/m^2^. One patient experienced serious adverse drug reactions (grade 3 panniculitis) on day 5 from the first drug administration. In particular, the patient complained about pain and heat sensation in her left lower leg. On day 8, she had difficulty walking and was admitted to the hospital. On day 9, a nodule changed to a tense blood blister and was broken spontaneously on the next day. This patient was withdrawn after the first dose of the first cycle, whereas all the remaining nine patients received more than one cycle of the study drug treatment. Of note, four patients met the criteria for a dose reduction in the first cycle due to neutropenia (*n* = 2), combination of neutropenia and maculo-papular rash (*n* = 1), or stomatitis (*n* = 1). The reasons leading to dose reductions in the first cycle are summarized in Supplemental Table 1.

### Safety

All enrolled patients received at least one study drug administration, so drug safety was analyzed in all 26 patients enrolled. NK105 was generally well tolerated. The most common hematologic toxicity was leukopenia (in 24 out of 26 patients) followed by neutropenia and lymphopenia (in 23 out of 26 patients each). The most frequent event at grade 3 or more was neutropenia (in 15 out of 26 patients) followed by leukopenia (in 12 out of 26 patients). G-CSF support was used for grade 4 neutropenia and leukopenia in one patient in Part II.

In contrast to the hematological toxicity manifestations, most nonhematological events were of grade 1 or 2. The most common event was peripheral sensory neuropathy (in 15 out of 26 patients), but in most cases it was of grade 1. Further details of this nonhematological toxicity are provided in the next paragraph. Even though one infusion reaction occurred, no patients experienced hypersensitivity during the study. Adverse drug reactions reported by more than 10 % patients are listed by dose level in Table 2.

**Table 2:** Hematological and nonhematological adverse drug reactions

	50 mg/m^2^ (*n* = 3)			65 mg/m^2^ (*n* = 3)			80 mg/m^2^ (*n* = 3)			100 mg/m^2^ (*n* = 7)			Dose-expansion phase (*n* = 10)		
	All	G3	G4	All	G3	G4	All	G3	G4	All	G3	G4	All	G3	G4
Hematological															
Leukocytopenia	2	0	0	3	0	0	3	2	0	6	3	0	10	5	2
Neutropenia	2	0	0	3	0	0	3	2	1	6	1	3	9	4	4
Lymphopenia	2	0	0	2	0	0	3	0	0	6	2	0	8	0	0
Erythropenia	0	0	0	0	0	0	1	1	0	6	0	0	3	0	0
Hemoglobin	2	0	0	0	0	0	2	1	0	7	1	0	7	0	0
Thrombocytopenia	0	0	0	0	0	0	1	0	0	1	0	0	2	0	0
Nonhematological															
Diarrhea	1	0	0	0	0	0	0	0	0	0	0	0	2	1	0
Nausea	2	0	0	0	0	0	0	0	0	3	0	0	1	0	0
Stomatitis	1	0	0	0	0	0	2	0	0	3	0	0	4	1	0
Fatigue	0	0	0	1	0	0	1	1	0	3	0	0	5	0	0
Edema peripheral	0	0	0	0	0	0	0	0	0	1	0	0	3	0	0
Pyrexia	1	0	0	1	0	0	1	0	0	1	0	0	1	0	0
Weight decreased	1	0	0	0	0	0	0	0	0	3	0	0	2	0	0
Decreased appetite	1	0	0	1	0	0	1	0	0	2	1	0	1	0	0
Arthralgia	0	0	0	0	0	0	0	0	0	0	0	0	3	0	0
Dysgeusia	1	0	0	0	0	0	1	0	0	0	0	0	3	0	0
Peripheral sensory neuropathy	1	0	0	1	0	0	1	0	0	5	1	0	7	0	0
Cough	1	0	0	1	0	0	0	0	0	0	0	0	1	0	0
Epistaxis	0	0	0	2	0	0	1	0	0	0	0	0	0	0	0
Alopecia	0	0	0	0	-	-	2	-	-	3	-	-	8	-	-
Dermatitis acneiform	0	0	0	0	0	0	0	0	0	2	0	0	2	0	0
Pruritus	0	0	0	0	0	0	1	0	0	1	0	0	2	0	0
Rash	0	0	0	0	0	0	1	0	0	1	0	0	4	0	0
Rash maculo-papular	1	0	0	1	0	0	1	0	0	1	0	0	3	0	0
Chemistry															
Albumin decreased	1	0	0	2	0	0	2	0	0	2	0	0	2	0	0
AST increased	2	0	0	0	0	0	0	0	0	1	0	0	1	0	0
ALT increased	2	0	0	1	0	0	0	0	0	1	0	0	2	0	0
g-GTP increased	0	0	0	0	0	0	0	0	0	1	0	0	3	0	0
ALP increased	1	0	0	0	0	0	0	0	0	1	0	0	2	0	0
Creatinine increased	0	0	0	1	0	0	0	0	0	1	0	0	1	0	0
Na decreased	0	0	0	0	0	0	0	0	0	2	1	0	1	0	0
CRP increased	1	0	0	1	0	0	1	0	0	2	0	0	4	1	0

Of the 16 patients treated in Part I, eight (50 %) experienced peripheral sensory neuropathy. This AE occurred in one of three patients at levels 2 (65 mg/m^2^) and 3 (80 mg/m^2^) in contrast to five out of seven patients treated at level 4 (100 mg/m^2^). One patient treated at level 4 (100 mg/m^2^) experienced grade 3 peripheral sensory neuropathy. No grade 4 peripheral sensory neuropathy was reported. Of the 10 patients treated in Part II, seven patients (70 %) experienced peripheral sensory neuropathy, including two patients that experienced grade 2 toxicity. One of the patients had an improvement of toxicity to grade 1 after a dose reduction, but later experienced its re-worsening to grade 2. Another patient had grade 2 toxicity until the termination of the treatment. Of the remaining five patients, four had grade 1 peripheral sensory neuropathy that persisted until treatment termination (Table 3). No grade 3 or higher peripheral sensory neuropathy cases have been observed.

**Table 3:** Severity of peripheral sensory neuropathy in each cycle. The first dose used in each cycle is indicated in the upper column for each patient

		Cycle																				
Patient ID		C1	C2	C3	C4	C5	C6	C7	C8	C9	C10	C11	C12	C13	C14	C15	C16	C17	C18	C19	C20	C21
BB-001	mg/m^2^	80	65	65	65																	
	Grade	G0	G0	G0	G0																	
BB-002	mg/m^2^	80	65	65	65	65	65	65														
	Grade	G0	G0	G0	G0	G0	G0	G0														
BB-003	mg/m^2^	80																				
	Grade	G0																				
BB-004	mg/m^2^	80	80	80	65	65	65	65	65	65	65											
	Grade	G1	G1	G1	G1	G1	G1	G2	G2	G2	G2											
BB-005	mg/m^2^	80	80	80	80	80	80	80	80	80	65	65	65	65	65							
	Grade	G1	G1	G1	G1	G1	G1	G1	G1	G2	G1	G1	G1	G1	G2							
BB-006	mg/m^2^	80	80	80	80																	
	Grade	G0	G1	G1	G1																	
BB-007	mg/m^2^	80	65	65	50	50	50	50	50	50	50	50	50	50	50	50	50	50	50	50	50	50
	Grade	G0	G0	G0	G0	G0	G1	G1	G1	G1	G1	G0	G0	G0	G0	G0	G0	G0	G0	G1	G1	G1
BB-008	mg/m^2^	80	65	50	50	50	50	50														
	Grade	G1	G1	G0	G0	G0	G0	G0														
BB-009	mg/m^2^	80	80	80	80	80	65	65	65	65	65	65	65	65								
	Grade	G0	G0	G0	G1	G1	G1	G1	G1	G1	G1	G1	G1	G1								
BB-010	mg/m^2^	80	80	80	80																	
	Grade	G1	G1	G1	G1																	

No treatment-related deaths occurred in the study. Six patients reported seven serious AEs. Six of the events were judged to be related to NK105 and included grade 4 hearing impairment, grade 3 ataxia, grade 2 infusion reaction, grade 3 decreased appetite, grade 3 panniculitis, and grade 3 hydronephrosis.

### Pharmacokinetics

**Fig. 1:**
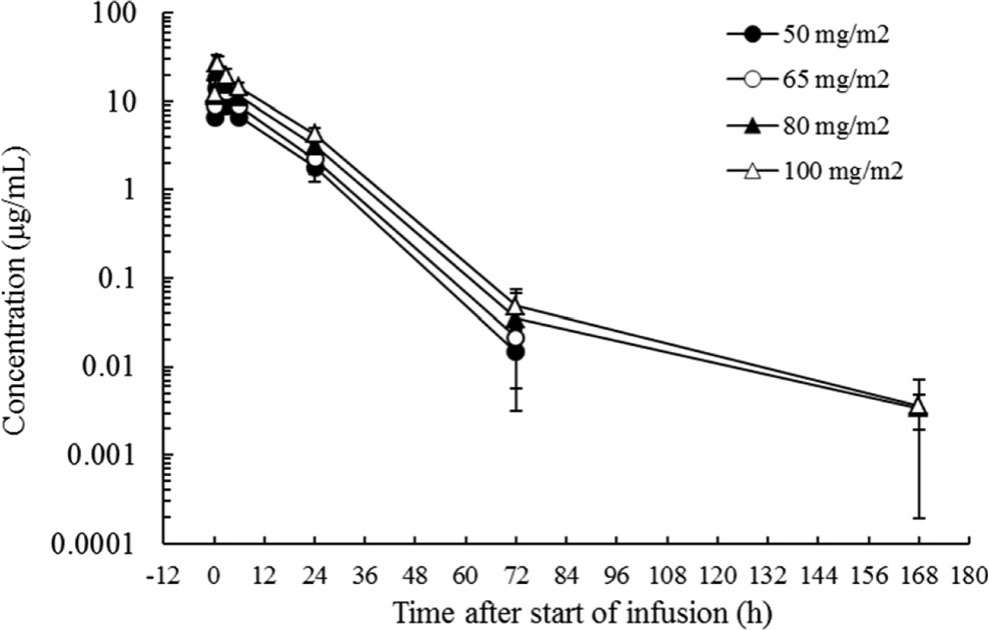
Paclitaxel plasma concentration time course following 30-min intravenous infusions of NK105 at 50–100 mg/m^2^ once weekly (1st cycle – 1st dose). Each data point represents the mean and S.D. of measurements from three patients except for data points for 100 mg/m^2^, which represent the mean and S.D. of measurements from six patients

The plasma concentrations of total PTX over time at each dose level are shown in Fig. 1, and PK parameters of NK105 are shown in Table 4. The plasma concentration of total PTX increased in a dose-dependent manner with its C_max_ and AUC_0-inf_ values being proportional to the administered dose. Its t_1/2_ value changed slightly with the increase in dose, while its CL_tot_, V_ss_, and MRT_0-inf_ values remained constant and independent of dose. When the plasma PTX concentration versus time profile after the third dose of the first cycle or after the first dose of the second cycle was compared with that after the first dose of the first cycle, PTX did not appear to accumulate in the plasma during once-weekly administration of NK105. Based on a comparison of the AUC_0-inf_ values of total and released PTX, approximately 5 % of total PTX in the plasma represented PTX released after administration of NK105 at each dose level (Table 5).

**Table 4:** Pharmacokinetic parameters of NK105

Dose		C_max_	T_max_	AUC_0-inf_	t_1/2_	CL_tot_	V_ss_	MRT_0-inf_
(mg/m^2^)	*n*	(μg/mL)	(h)	(μg• h/mL)	(h)	(mL/h/m^2^)	(mL/m^2^)	(h)
50	3	14.2	0.69	176	8.84	286	3140	11.0
		± 2.0	± 0.27	± 17	± 3.26	± 27	±160	± 0.6
65	3	18.3	0.71	231	10.3	283	3070	10.8
		± 1.3	± 0.25	± 18	± 2.7	± 22	± 360	± 0.5
80	3	22.0	0.80	302	12.7	284	3080	11.2
		± 3.3	± 0.30	± 104	± 0.8	± 82	± 590	± 2.0
100	6	27.6	0.80	390	12.3	262	3150	12.0
		± 5.3	± 0.30	± 64	± 1.3	± 39	± 580	± 0.9

**Table 5:** AUC of total and released paclitaxel

		AUC_0-inf_ (μg• h/mL)			
		50 mg/m^2^	65 mg/m^2^	80 mg/m^2^	100 mg/m^2^
Total PTX	Mean	176	231	302	390
	S.D.	17	18	104	64
Released PTX	Mean	9.83	16.2	20.0	16.7
	S.D.	5.04	5.6	9.7	1.6

### Tumor response

**Fig. 2:**
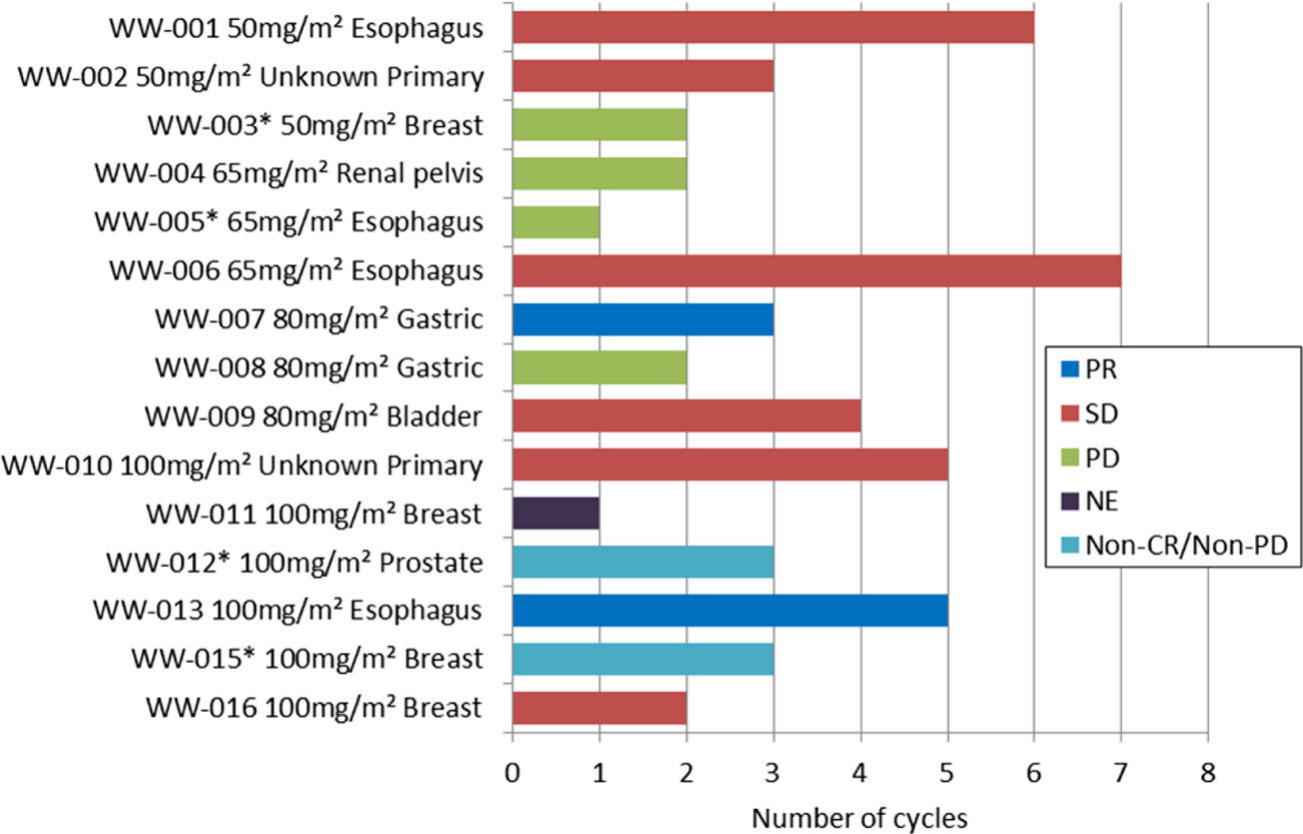
Efficacy of NK105 in the dose-escalation cohort (Part I). Both best overall responses and the number of cycles received by each of the 15 patients are indicated. Asterisk (*) indicates the patient who did not have any measurable lesion(s) as defined by the RECIST guideline version 1.1

Twenty-five patients (15 from Part I and 10 from Part II) were evaluated for the tumor response. Four out of 15 patients in Part I did not have any measureable lesions. Figure 2 shows the numbers of treatment cycles that patients received and corresponding efficacy assessments according to RECIST. All patients that received three or more treatment cycles achieved PR or stable disease (SD).

**Fig. 3:**
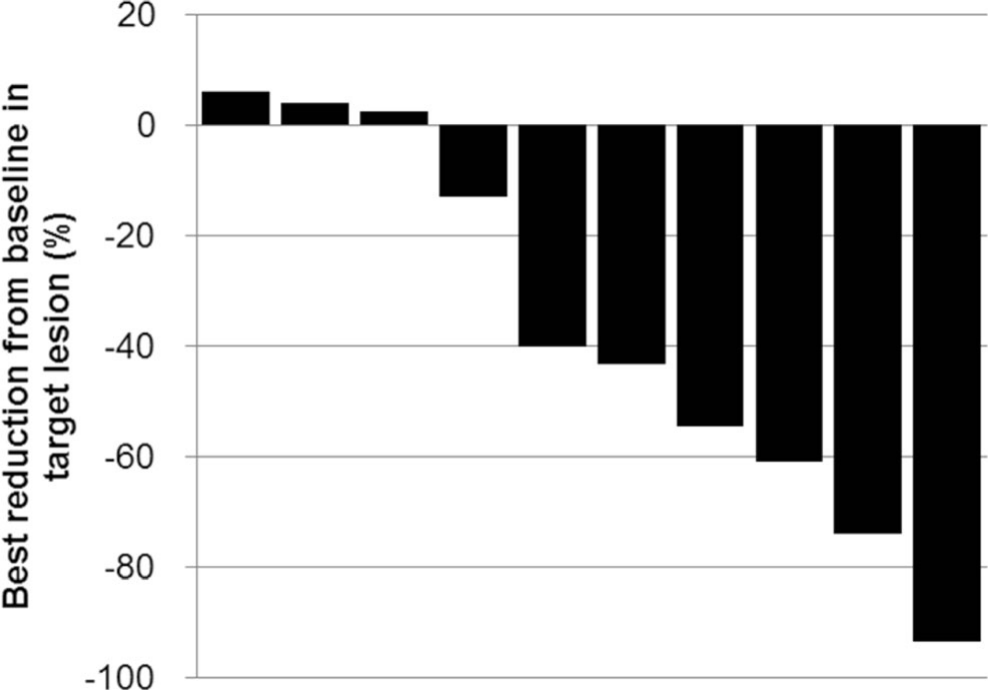
A waterfall plot of the best response in breast cancer patients with RECIST-evaluable disease in the expansion cohort (Part II)

Of the 10 breast cancer patients in Part II, six achieved PR and four achieved SD. The ORR was 60.0 % (95 % CI 26.2–87.8 %), and the disease control rate (percentage of patients with CR, PR, or SD) was 100.0 % (95 % CI 69.2–100.0 %). Four patients received more than 10 cycles of NK105. Figure 3 shows the greatest tumor size reductions as percentages of baseline plotted in a waterfall format. Seven out of 10 patients in Part II had partial reductions of their target lesions after receiving NK105. Seven patients had to reduce their doses to 65 mg/m^2^ or even to 50 mg/m^2^ according to the abovementioned criteria or at the investigator’s discretion, and continuous disease control was observed even after the dose reductions.

## Discussion

PTX is one of the key chemotherapeutic agents used widely in current medical practice. However, its therapeutic benefit is limited by its poor water solubility, which complicates its dosing procedures and leads to additional toxicity. The conventional formulation of PTX requires reconstitution with Cremophor EL, which is considered the main cause of hypersensitivity reaction during PTX infusions. To prevent this severe form of allergy, the conventional PTX formulation must always be administered with an antiallergic premedication containing a corticosteroid and an antihistamine. Therefore, a novel PTX formulation that can be administered without the use of such vehicles and premedication is desirable. Recently, nanoparticle albumin-bound PTX (nab-PTX, Abraxane®), which can be infused without an antiallergic premedication to prevent hypersensitivity, has been introduced into clinical use [14]. However, nab-PTX contains human albumin, suggesting a potential risk of infection.

NK105 is a novel drug delivery system formulation of PTX composed of PTX-encapsulated nanoparticle micelles. NK105 can be administered without an antiallergic premedication and does not need not to be solubilized in vehicles such as Cremophor EL, because it is soluble in aqueous vehicles, such as the one used in the present study (5 % glucose solution). In addition, NK105 can be administered intravenously over 30 min. It is therefore expected that such features of NK105 would considerably reduce the burden on medical staff and patients. Furthermore, NK105 is known to have a unique PK profile, different from that of clinically available PTX formulations, which leads to a greater tumor response and weaker propensity to cause peripheral neurotoxicity [9].

When NK105 was administered once weekly at 50–100 mg/m^2^ as a 30-min infusion, the plasma concentration of total PTX increased in a dose-dependent manner, and its C_max_ and AUC_0-inf_ were directly proportional to the administered dose. The t_1/2_ value also tended to increase with the increase in dose. However, this tendency was likely to reflect unmeasurably low plasma PTX concentrations at 168 h post-dose in two patients treated at 50 mg/m^2^ and one patient treated at 65 mg/m^2^ that would shorten the mean t_1/2_ at these dose levels. Therefore, the PTX t_1/2_ value after administration of NK105 should not change considerably with an increase in dose. The CL_tot_, V_ss_, and MRT_0-inf_ values remained constant and independent of dose. Thus, NK105 exhibited linear PK at doses of 50–100 mg/m^2^ in patients. The PTX AUC_0-inf_ value at 100 mg/m^2^ NK105 was 390 μg•h/mL, and V_ss_ was 3150 mL/m^2^. In comparison to the corresponding values of the conventional PTX preparation (Taxol®; AUC_0-inf_ 7.88 μg•h/mL, V_ss_ 74.7 L/m^2^ [15]), a 50-fold greater AUC_0-inf_ value and about 25-fold smaller V_ss_ were observed for NK105. This indicates that encapsulation of PTX in the NK105 polymer can produce a much longer plasma retention of the drug in humans, as expected from nonclinical findings [4]. In the present study, we examined the plasma PK PTX released from NK105. At the four dose levels examined, about 5 % of total PTX present in the micelles was released free into the plasma in terms of the AUC_0-inf_ values. These data suggest that the majority of the plasma PTX remains within the nanomicelles, and that this is a likely mechanism for the sustained plasma retention of PTX after administration of NK105.

Of the 15 evaluable patients treated in Part I, two and six patients, respectively, achieved best overall PR and SD responses. Of the two patients who achieved PR, one was treated at 80 mg/m^2^ for gastric cancer, and the other was treated at 100 mg/m^2^ for esophageal/oral floor cancer. NK105 may be active against these malignancies, which are included in the currently approved indications for PTX. In patients with advanced breast cancer (Part II), a preferable ORR of 60 % (6/10) was observed. Efficacy of Taxol® at six weekly doses of 100 mg/m^2^ followed by a two-week rest in patients with advanced breast cancer was reported as ORR of 44.9 % (31/69) [16]. Thus, NK105 may be as effective against breast cancer as conventional PTX, although the small sample size prevents us from making a definite conclusion.

Currently available PTX formulations are known to frequently cause peripheral neurotoxicity that can become severe enough to cause difficulty doing fine motor tasks and walking. Thus, it considerably impairs patient’s quality of life. In a phase II study of weekly Taxol® conducted in Japan, 17 (24.6 %) and 4 (5.8 %) of 69 patients, respectively, developed grade 2 and 3 neuropathy [16]. In the present study, there was no grade 3 or more peripheral sensory neuropathy in the Part II cohort. In addition, although two out of the 10 patients experienced grade 2 peripheral sensory neuropathy, the severity of this toxicity was mostly grade 0 or 1 across all treatment cycles. Further clarification is necessary of whether NK105 indeed causes less peripheral neurotoxicity than conventional PTX.

In Part I, the recommended dose of weekly administration of NK105 was determined to be 80 mg/m^2^ through a consultation with an independent data monitoring committee. In part II, additional 10 patients were enrolled to examine preliminary safety and efficacy of weekly NK105 at the recommended dose. Except for one patient who experienced a serious AE (panniculitis) after the first dose, the remaining nine patients completed the first cycle, six patients received more than seven cycles, and four patients received more than 10 cycles. Furthermore, although the number of patients was limited, the preferable tumor response (ORR 60 %) was observed. Considering these results, we concluded that weekly NK105 was well tolerated and had desirable antitumor activity, so a further investigation for patients with ABC was warranted. One of the strategies to show clinical usefulness of NK105 is to verify its non-inferiority to conventional PTX in terms of the efficacy endpoint showing a preferable safety profile and clinical convenience, including absence of the premedication requirement and shorter administration time. Weaker peripheral neurotoxicity in NK105 has been suggested from pre-clinical studies and a published phase II study [9]. At the same time, the high frequency of grade ≥ 3 neutropenia and corresponding frequent dose reductions or dose delays in earlier treatment phases by NK105 at 80 mg/m^2^ were the main concerns. In the first cycle, four patients met the criteria for a dose reduction, mostly due to neutropenia, and grade ≥ 3 neutropenia that occurred in eight out of 10 patients (80 %) throughout the treatment courses. In a phase II study of Taxol®, grade ≥ 3 neutropenia manifested in 26 out of 69 breast cancer patients (37.7 %) who received six weekly doses of 100 mg/m^2^ [16]. Based on the comparison with the results of that Taxol® study, NK105 at 80 mg/m^2^ per week may be more likely to induce grade ≥ 3 neutropenia than conventional PTX. Taken these safety and efficacy aspects into consideration, we finally decided that the dose of weekly NK105 for the subsequent Phase III study to examine non-inferiority of NK105 to conventional PTX in patients with advanced breast cancer would be set to 65 mg/m^2^, i.e., one level lower than the recommended dose determined in the dose-escalation phase. A multinational phase III study comparing NK105 and conventional PTX in patients with metastatic or recurrent breast cancer is currently underway (ClinicalTrials.gov identifier: NCT01644890).

## Electronic supplementary material

Supplemental Table 1 (DOCX 26 kb)

## References

[1] Carney DN (1996). Chemotherapy in the management of patients with inoperable non-small cell lung cancer. Semin Oncol.

[2] Rowinsky EK, Cazenave LA, Donehower RC (1990). Taxol: a novel investigational antimicrotubule agent. J Natl Cancer Inst.

[3] Crown J, O’Leary M (2000). The taxanes: an update. Lancet.

[4] Hamaguchi T, Matsumura Y, Suzuki M, Shimizu K, Goda R, Nakamura I, Nakatomi I, Yokoyama M, Kataoka K, Kakizoe T (2005). NK105, a paclitaxel-incorporating micellar nanoparticle formulation, can extend in vivo antitumor activity and reduce the neurotoxicity of paclitaxel. Br J Cancer.

[5] Matsumura Y, Maeda H (1986). A new concept for macromolecular therapeutics in cancer chemotherapy: mechanism of tumoritropic accumulation of proteins and the antitumor agent smancs. Cancer Res.

[6] Maeda H, Wu J, Sawa T, Matsumura Y, Hori K (2000). Tumor vascular permeability and the EPR effect in macromolecular therapeutics: a review. J Control Release.

[7] Nakanishi T, Fukushima S, Okamoto K, Suzuki M, Matsumura Y, Yokoyama M, Okano T, Sakurai Y, Kataoka K (2001). Development of the polymer micelle carrier system for doxorubicin. J Control Release.

[8] Hamaguchi T, Kato K, Yasui H, Morizane C, Ikeda M, Ueno H, Muro K, Yamada Y, Okusaka T, Shirao K, Shimada Y, Nakahama H, Matsumura Y (2007). A phase I and pharmacokinetic study of NK105, a paclitaxel-incorporating micellar nanoparticle formulation. Br J Cancer.

[9] Kato K, Chin K, Yoshikawa T, Yamaguchi K, Tsuji Y, Esaki T, Sakai K, Kimura M, Hamaguchi T, Shimada Y, Matsumura Y, Ikeda R (2012). Phase II study of NK105, a paclitaxel-incorporating micellar nanoparticle, for previously treated advanced or recurrent gastric cancer. Investig New Drugs.

[10] Cardoso F, Costa A, Norton L (2014). ESO-ESMO 2nd international consensus guidelines for advanced breast cancer (ABC2). Ann Oncol.

[11] Seidman AD, Berry D, Cirrincione C, Harris L, Muss H, Marcom PK, Gipson G, Burstein H, Lake D, Shapiro CL, Ungaro P, Norton L, Winer E, Hudis C (2008). Randomized phase III trial of weekly compared with every-3-weeks paclitaxel for metastatic breast cancer, with trastuzumab for all HER-2 over-expressors and random assignment to trastuzumab or not in HER-2 nonoverexpressors: final results of Cancer and Leukemia Group B protocol 9840. J Clin Oncol.

[12] Clopper CJ, Pearson ES (1934). The use of confidence or fiducial limits illustrated in the case of the binomial. Biometrika.

[13] Lachin JM (2000). Biostatistical Methods: The Assessment of Relative Risks.

[14] Gupta N, Hatoum H, Dy GK (2014). First line treatment of advanced non-small-cell lung cancer - specific focus on albumin bound paclitaxel. Int J Nanomedicine.

[15] Tamura T, Sasaki Y, Nishiwaki Y, Saijo N (1995). Phase I study of paclitaxel by three-hour infusion: hypertension just after infusion is one of the major dose-limiting toxicities. Jpn J Cancer Res.

[16] Horiguchi J, Rai Y, Tamura K (2009). Phase II study of weekly paclitaxel for advanced or metastatic breast cancer in Japan. Anticancer Res.

