# Supplementary material for: Phase I study of NK105, a nanomicellar paclitaxel formulation, administered on a weekly schedule in patients with solid tumors
Source: Invest New Drugs. 2016 Sep 5;34(6):750–9. doi: 10.1007/s10637-016-0381-4 (PMC5099351; doi:10.1007/s10637-016-0381-4)
Supplement: Supplementary file 1 — (DOCX 26 kb) [file 10637_2016_381_MOESM1_ESM.docx]

**Supplemental Table 1** List and reasons for dose reductions in the first cycle

| Phase | Dose | Patient ID | Reasons |
| --- | --- | --- | --- |
| Dose-escalation phase | 100 mg/m^2^ | WW-011 | ANC decreased (DLT) |
|  |  | WW-015 | ANC decreased (duration of delay) |
| Dose-expansion phase | 80 mg/m^2^ | BB-001 | Stomatitis (DLT) |
|  |  | BB-002 | ANC decreased (DLT) |
|  |  | BB-007 | ANC decreased, dermatitis (duration of delay) |
|  |  | BB-008 | ANC decreased (duration of delay) |
